# Neuroinflammation as a failure of allostatic integrity: a hierarchical-circular model of biological memory for pathogenesis in neurodegenerative and neuropsychiatric diseases

**DOI:** 10.3389/fnsys.2026.1888548

**Published:** 2026-07-10

**Authors:** Samuel Ruesga Mundo

**Affiliations:** 1 Hospital de Especialidades del Centro Médico Nacional de Occidente, Instituto Mexicano del Seguro Social (IMSS), Guadalajara, Jalisco, Mexico; 2 Department of Psychiatry, Regional General Hospital 180, IMSS, Tlajomulco de Zúñiga, Jalisco, Mexico

**Keywords:** allostasis, allostatic integrity, biological memory, hierarchical-circular model, neurodegeneration, neuroinflammation, neuropsychiatry, PINE system

## Abstract

**Introduction:**

Chronic neuroinflammation is a hallmark of neurodegenerative diseases (Alzheimer’s disease, Parkinson’s disease) and neuropsychiatric disorders (major depression, schizophrenia). However, current models fail to explain why some individuals develop persistent neuroinflammation while others maintain homeostasis, nor why similar inflammatory pathology produces such diverse clinical phenotypes.

**Hypothesis:**

We propose that pathological neuroinflammation emerges from a failure of allostatic integrity—the capacity of the organism to maintain adaptive circular information flow across five interconnected levels: (1) genetic architecture and morphogenetic programming, (2) epigenetic molecular memory, (3) allostatic load and systemic physiological adaptation, (4) the psychoneuroimmuneendocrine (PINE) network, and (5) interoceptive-neuronal integration. When this circular flow is disrupted, maladaptive stable states become entrenched, perpetuating chronic inflammation.

**Development of the hypothesis:**

Synthesizing evidence from longitudinal and mechanistic studies (with foundational older works cited where necessary), we discuss how: (a) early-life stress epigenetically programs inflammatory reactivity; (b) elevated allostatic load predicts chronic neuroinflammation; (c) PINE network dysregulation perpetuates pro-inflammatory signaling; and (d) interoceptive dysfunction may prevent the downregulation of inflammation. Allostatic integrity is introduced as a dynamic systems-level property hypothesized to moderate the relationship between inflammatory pathology and clinical expression.

**Testable predictions:**

The framework generates falsifiable predictions: (1) composite indices of allostatic integrity will outperform single biomarkers in predicting transition to chronic neuroinflammation; (2) multidomain interventions targeting multiple levels will produce multiplicative (synergistic) effects; (3) patients with similar inflammatory profiles but contrasting allostatic integrity will show markedly different clinical trajectories; and (4) improvements in allostatic integrity will correlate with reduced neuroinflammation independent of direct anti-inflammatory therapies.

**Conclusion:**

The Hierarchical-Circular Model reframes chronic neuroinflammation not as a linear cascade but as a potential systemic failure of biological memory and allostatic integrity. Pending empirical validation, this framework may offer a conceptual basis for biomarker development, multidomain prevention, and personalized treatment strategies.

## 1 Introduction

Neuroinflammation—characterized by microglial activation, astrogliosis, elevated pro-inflammatory cytokines (IL-1β, IL-6, TNF-*α*), and oxidative stress—has emerged as a common pathological feature across traditionally distinct diagnostic categories. It is central to neurodegenerative diseases such as Alzheimer’s disease (AD), Parkinson’s disease (PD), and Huntington’s disease (Glass et al., 2010; Heneka et al., 2015), and increasingly recognized in neuropsychiatric conditions including major depressive disorder (MDD) and schizophrenia (Miller and Raison, 2016; Goldsmith et al., 2016).

Despite this convergence, three fundamental paradoxes remain unresolved.

### 1.1 Paradox 1: pathology-expression dissociation

Many individuals with elevated neuroinflammatory markers remain cognitively and behaviorally intact, while others with modest inflammation exhibit severe impairment (Bennett et al., 2016; Jack et al., 2019). This dissociation suggests that neuroinflammation is neither necessary nor sufficient for clinical disease—yet current models cannot fully explain why.

### 1.2 Paradox 2: early-life determinants of late-life inflammation

Adverse childhood experiences and prenatal stress are consistently associated with elevated inflammatory markers decades later (Danese et al., 2007; Slopen et al., 2010; Ruesga-Mundo et al., 2025). How early-life events become biologically embedded to produce chronic neuroinflammation in late life requires further elucidation.

### 1.3 Paradox 3: heterogeneous treatment responses

Anti-inflammatory strategies—from NSAIDs to cytokine inhibitors—have produced inconsistent results across trials (Aisen et al., 2003; ADAPT Research Group et al., 2008; Rivers-Auty et al., 2020; Kappelmann et al., 2018). Some patients respond dramatically; others show no benefit (Raison et al., 2013; Kappelmann et al., 2018). This heterogeneity suggests that neuroinflammation may not be a single pathological entity but rather an emergent property of complex, multi-level systems.

These paradoxes indicate a need for integrative frameworks that transcend linear, reductionist models. In this article, we propose a Hierarchical-Circular Model of Biological Memory that reframes chronic neuroinflammation as a potential failure of allostatic integrity—the hypothesized capacity of the organism to maintain adaptive circular information flow across multiple levels of biological organization.

## 2 Methods for evidence synthesis

This article presents a narrative (conceptual) synthesis, not a systematic review or meta-analysis. No formal quantitative pooling was performed in this manuscript, except where explicitly noted as imported from external published meta-analyses.

### 2.1 Search strategy

Databases searched included PubMed, Google Scholar, and Web of Science. The primary time range was 2015–2026, with landmark older citations included where necessary for foundational concepts (McEwen and Stellar, 1993; Hardy and Higgins, 1992; Ader and Cohen, 1975).

### 2.2 Search terms

Combinations of the following terms were used: “neuroinflammation AND allostatic load,” “epigenetics AND early-life stress,” “PINE network,” “psychoneuroimmunology,” “interoception AND inflammation,” “biological memory,” “allostatic integrity.”

### 2.3 Inclusion logic

Studies were included if they (a) addressed at least one of the five levels of the proposed model, (b) were published in peer-reviewed journals, and (c) provided mechanistic, longitudinal, or interventional data relevant to neuroinflammation.

### 2.4 Handling of conflicting evidence

Contradictory findings (e.g., heterogeneity in anti-inflammatory trials) are discussed where relevant. The model explicitly aims to explain such heterogeneity rather than ignore it (Table 1).

**Table 1:** Comparison between the present hypothesis and theory article and a systematic review.

Aspect	Present article (hypothesis and theory)	Systematic review (for comparison)
Objective	Develop an integrative theoretical framework and generate testable hypotheses	Answer a specific clinical question through quantitative synthesis
Search strategy	Narrative, with flexible criteria to identify key concepts	Systematic, with pre-registered protocol (PROSPERO) and exhaustive search strategy
Inclusion criteria	Broad: any study addressing at least one level of the proposed model	Strict: pre-defined PICO with explicit eligibility criteria
Evidence synthesis	Qualitative (conceptual), narrative integration of findings	Quantitative (meta-analysis) where possible, with heterogeneity assessment
Risk of bias assessment	Not performed systematically; contradictory findings are discussed	Standardized tools (Cochrane ROB, Newcastle-Ottawa)
PRISMA statement	Not applicable	Required
Registration	Not registered	PROSPERO or other registry

## 3 The insufficiency of purely linear models of neuroinflammation

### 3.1 What linear cascade models are we criticizing?

By “linear cascade models,” we refer to frameworks that postulate predominantly unidirectional or feedforward pathways. Examples include the amyloid cascade hypothesis in its original formulation (Hardy and Higgins, 1992), or simplified models of microglial activation where trigger → cytokine release → neuronal damage, without adequate incorporation of feedback loops or multi-level regulation. We acknowledge that many contemporary models have moved beyond strict linearity. Our critique targets simplified formulations and the absence of integrated multi-level circular architectures, not the entirety of the field.

### 3.2 Limitations of linear approaches

While linear cascade models have generated valuable mechanistic insights, they face critical limitations:

**Table:** 

Limitation	Explanation
Underappreciated bidirectionality	Some models assume inflammation → damage, with less emphasis on how damage → inflammation
Single-level focus	Many models examine molecular/cellular events without integrating systemic physiology, psychology, or development
Static rather than dynamic	Some formulations treat inflammation as a state, not as an emergent property of circular feedback
Limited incorporation of individual history	Many models rarely incorporate early-life programming, epigenetic embedding, or cumulative stress

### 3.3 Relationship to existing frameworks

The proposed model builds upon—and is not a replacement for—several established frameworks.

- Psychoneuroimmunology (PNI): This field established the foundational principle of bidirectional brain-immune communication (Ader and Cohen, 1975; Ader, 2006). What PNI lacks is a hierarchical organization across multiple levels of biological memory and explicit treatment of stability states. The present model adds a formal hierarchical-circular architecture that specifies how bidirectional signals become entrenched as maladaptive stable states.
- Allostatic load model (McEwen and Stellar, 1993; McEwen, 1998). This formulation introduced allostatic load and allostatic overload as cumulative wear and tear from chronic stress adaptation (McEwen, 1998). What the allostatic load model lacks is explicit integration with epigenetic memory, the PINE network as a structured level, and interoceptive regulation. The present model adds allostatic integrity as a complementary dynamic construct (adaptive capacity vs. cumulative damage) and specifies five levels through which allostatic processes operate.
- Predictive coding/active inference/interoception (Friston, 2010; Seth and Friston, 2016; Barrett and Simmons, 2015; Khalsa et al., 2018). These frameworks have revolutionized understanding of interoception as inference about internal states (Friston, 2010). What they lack is direct application to chronic neuroinflammation and integration with peripheral physiology and epigenetic memory. The present model bridges interoceptive predictive coding with systemic physiology and molecular memory.
- Network medicine (Barabási et al., 2011). Network medicine conceptualizes disease as emergent from disrupted network properties (Barabási et al., 2011). What it lacks is a specific hierarchical architecture for neuroinflammation. The present model provides a domain-specific instantiation of network principles for neuroinflammation.

*What the hierarchical-circular model adds beyond existing frameworks (summary)*: (1) explicit circular causality across five specified levels, (2) the “Signal → Plasticity → Stable State” principle as a unifying dynamic, (3) interoception as a top-level regulator, and (4) testable predictions about intervention synergy and biomarker performance.

## 4 The hierarchical-circular model of biological memory applied to neuroinflammation

### 4.1 The unifying principle: “signal → plasticity → stable state”

At the core of our model lies a principle applicable across all levels of biological organization: Signal → Plasticity → Stable State. Biological systems continuously receive signals from internal and external environments, undergo plastic changes in response, and settle into stable states that encode the history of prior signals. This dynamic operates iteratively, such that each stable state becomes the basis for responding to subsequent signals—establishing circular causality.

**Figure 1:**
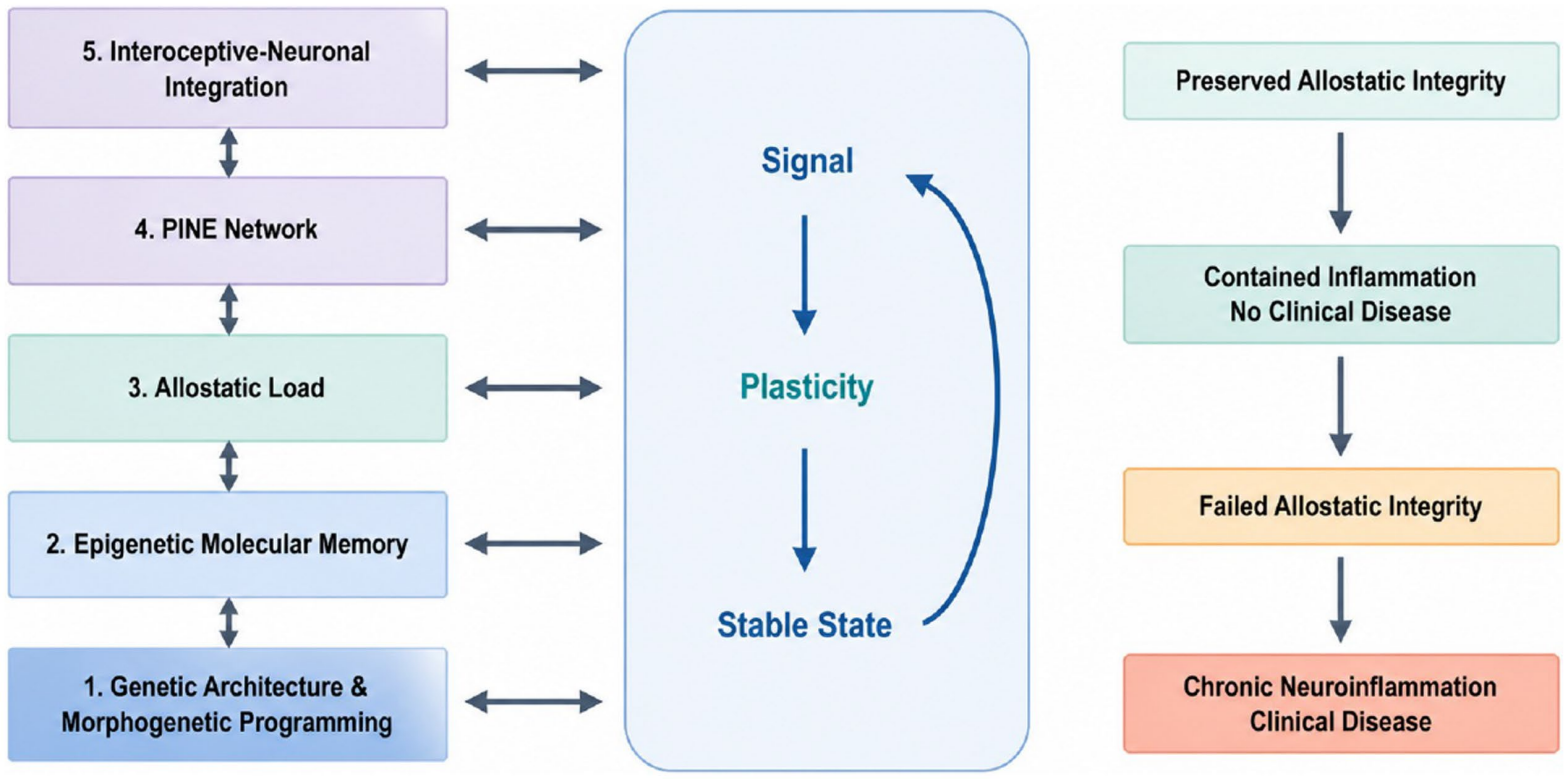
Hierarchical-circular model of allostatic integrity. The model proposes five interconnected biological levels arranged in hierarchical order: genetic architecture and morphogenetic programming, epigenetic molecular memory, allostatic load, the PINE network, and interoceptive-neuronal integration. Thes’e levels interact bidirectionally with a central circular regulatory loop organized as signal, plasticity, and stable state. Preserved allostatic integrity is associated with contained inflammation and absence of clinical disease, whereas failed allostatic integrity is associated with chronic neuroinflammation and clinical disease.

When this circular dynamic functions adaptively, the organism maintains allostatic integrity. When disrupted at any level, maladaptive stable states may become entrenched, and chronic neuroinflammation may emerge as a systems-level failure (Figure 1).

### 4.2 Level 1: genetic architecture and morphogenetic programming

The first level encompasses the genetic blueprint that establishes baseline inflammatory reactivity. Genetic factors, including polymorphisms in genes encoding pro-inflammatory cytokines (IL-6, TNF-*α*, IL-1β) and stress-response proteins (FKBP5, NR3C1), influence individual differences in neuroinflammatory susceptibility (Binder, 2009; Hohmann et al., 2013; Ruesga-Mundo et al., 2026).

Additionally, the apolipoprotein E ε4 (APOE4) allele—the strongest genetic risk factor for late-onset Alzheimer’s disease—exemplifies how genetic variation shapes neuroinflammatory vulnerability. APOE4 has been shown to enhance microglial inflammatory signaling, alter neuronal lipid metabolism, and increase neuronal network activity, thereby establishing a pro-inflammatory baseline that interacts with other levels of the model (Verduzco Espinoza et al., 2025).

Crucially, genetic architecture interacts with morphogenetic programming—the processes by which genetic information guides tissue organization during embryogenesis. *Important caveat:* This developmental hypothesis is speculative and requires direct empirical testing, which is currently lacking. Morphogenetic programming may constrain the range of possible inflammatory responses later in life but does not determine them. As previously articulated:

“Morphogenetic fields, defined by signaling gradients during gastrulation and organogenesis, do not persist as dynamic entities in the adult; however, their action leaves a structural and epigenetic footprint that conditions the functional organization of neuroendocrine, immune, and behavioral systems throughout life” (Ruesga-Mundo et al., 2026).

The Spemann-Mangold organizer and the notochord establish the basic architecture of the hypothalamic–pituitary–adrenal (HPA) axis, the autonomic nervous system, and immune cell precursors. This developmental programming may set the range within which inflammatory responses can operate across the lifespan (Gilbert, 2016; Ruesga-Mundo, 2025a).

### 4.3 Level 2: epigenetic molecular memory

Independent meta-analyses have demonstrated that early-life stress is associated with significant DNA methylation changes in stress-related genes (NR3C1, SLC6A4, FKBP5) (Park et al., 2019; Cecil et al., 2020). Consistent with this, our systematic review found a pooled standardized mean difference of −0.72 (95% CI: −1.15 to −0.29; *p* = 0.001) (Ruesga-Mundo et al., 2026). This meta-analysis included k = 7 studies examining stress-related gene expression or DNA methylation changes. Heterogeneity was moderate (I^2^ = 54%), reflecting differences in tissue type (e.g., buccal cells, blood, post-mortem brain) and specific loci examined. Subgroup analyses revealed slightly larger effect sizes in studies using post-mortem brain tissue (SMD = −0.89; 95% CI: −1.34 to −0.44) compared to those using peripheral blood (SMD = −0.61; 95% CI: −1.02 to −0.20), although this difference did not reach statistical significance (p for interaction = 0.18). The full systematic review included 58 studies total (30 in quantitative synthesis), with details of sample characteristics, risk of bias assessment (using RoB 2, ROBINS-I, and Newcastle-Ottawa scales), and GRADE quality evaluation reported in the original publication (Ruesga-Mundo et al., 2026) and its supplementary material. These epigenetic marks persist for years and predict inflammatory reactivity to subsequent challenges. Importantly, epigenetic marks can be modified by ongoing experiences—including psychosocial interventions, metabolic changes, and pharmacological agents (Feng et al., 2025)—opening possibilities for intervention.

### 4.4 Level 3: allostatic load and systemic physiological adaptation

The third level encompasses the cumulative physiological consequences of repeated adaptation to environmental demands. Allostatic load—the “wear and tear” on the body resulting from sustained activation of stress-response systems (McEwen and Stellar, 1993; McEwen, 1998)—is typically measured through composite indices integrating cardiovascular, metabolic, and inflammatory markers (Guidi et al., 2021).

Longitudinal studies have consistently demonstrated that elevated allostatic load prospectively predicts chronic inflammation, cognitive decline, and all cause dementia (Ruan et al., 2025; Souza-Talarico et al., 2025). Importantly, some evidence suggests allostatic load effects may be independent of classical pathology (e.g., amyloid burden), indicating that systemic physiology may contribute to neuroinflammation through pathways distinct from traditional cascades (Souza-Talarico et al., 2025).

As previously proposed, resilience may emerge as an observable manifestation of efficiently calibrated allostatic systems, evidenced by multisystem coordination, predictive regulation, and efficient recovery from challenges (Ruesga-Mundo, 2025b). Within the present framework, allostatic load (cumulative wear) and allostatic integrity (dynamic adaptive capacity) are complementary constructs.

### 4.5 Level 4: the psychoneuroimmuneendocrine (PINE) network

The fourth level captures the dynamic interactions among psychological processes, neurological function, endocrine signaling, and immune activity—what we have termed the PINE network (Ruesga-Mundo, 2025c; Ruesga-Mundo et al., 2026).

Psychological stress activates the HPA axis and sympathetic nervous system, triggering cortisol and catecholamine release that modulate immune cell trafficking and cytokine production (Dantzer et al., 2008; Slavich and Irwin, 2014). Chronic stress promotes a pro-inflammatory phenotype characterized by elevated IL-6, TNF-*α*, and CRP, which can compromise the blood–brain barrier and promote neuroinflammation (Feng et al., 2025; Tyagi and Bartley, 2025).

Conversely, peripheral inflammation signals the brain through multiple pathways—vagal afferents, circulating cytokines, endothelial activation—inducing sickness behaviors, cognitive deficits, and neuroinflammation. This bidirectionality is central to the circular model: perturbations at any point in the PINE network propagate bidirectionally, potentially amplifying maladaptive states.

### 4.6 Level 5: interoceptive-neuronal integration

The fifth and highest level concerns the integration of interoceptive signals—sensory information about the internal state of the body—with neuronal dynamics across large-scale brain networks. Interoception provides the foundation for emotional experience, self-awareness, and adaptive regulation of internal states (Barrett and Simmons, 2015; Khalsa et al., 2018; Friston, 2010; Seth and Friston, 2016).

Recent advances reveal that interoceptive processing relies on intrinsic neural timescales—the temporal windows over which neurons integrate information. In behavioral-variant frontotemporal dementia, altered intrinsic neural timescales of interoceptive processing have been demonstrated, with disruptions in allostatic-interoceptive networks that support adaptive regulation (Hazelton et al., 2025).

Critically, interoceptive dysfunction may perpetuate neuroinflammation through a bidirectional loop: poor interoceptive accuracy → misregulation of physiological states → sustained inflammatory signaling → further interoceptive impairment. This loop exemplifies the circular dynamics central to our model.

### 4.7 Circular dynamics and bidirectional propagation across levels

A defining feature of our model is the emphasis on bidirectional propagation of signals across levels. Perturbations at any level can propagate upward, downward, and laterally:

**Table:** 

Propagation direction	Example
Upward	Genetic variant (Level 1) → epigenetic susceptibility (Level 2) → allostatic load accumulation (Level 3) → PINE dysregulation (Level 4) → interoceptive dysfunction (Level 5)
Downward	Chronic psychological stress (Level 4) → elevated allostatic load (Level 3) → epigenetic modifications (Level 2) → altered gene expression (Level 1)
Lateral	Neuroimmune activation (Level 4) → directly influences interoceptive networks (Level 5) while simultaneously modulating allostatic load (Level 3)

This circular architecture may help explain why individuals with similar inflammatory profiles follow divergent clinical trajectories. When the system maintains allostatic integrity—the capacity for adaptive circular information flow—inflammation may be contained without clinical expression. When allostatic integrity fails, maladaptive stable states become entrenched (Table 2).

**Table 2:** Distinction from related constructs (hypothesized).

Construct	Definition	How allostatic integrity differs (hypothesized)
Allostatic load	Cumulative wear and tear (higher = worse)	Cumulative adaptive capacity (higher = better); complementary construct
Cognitive reserve	Neural buffering against pathology	Multisystem (includes peripheral physiology, epigenetics, interoception)
Frailty index	Deficit accumulation	Specifically captures adaptive circular flow
Resilience (traditional)	Outcome of successful adaptation	The systems property hypothesized to produce resilience

### 4.8 Allostatic integrity as an emergent systems property

Allostatic integrity is the central construct of our model. It represents the hypothesized dynamic adaptive capacity of the organism to maintain circular information flow across Levels 1–5. It is proposed to be:

- Dynamic (not static; changes across the lifespan).
- Multisystem (not reducible to any single biomarker).
- Emergent (arises from interactions, not from any component alone).
- Moderating (may determine the clinical impact of neuroinflammation).

*Note on discriminant validity*: The discriminant validity of allostatic integrity relative to neighboring constructs is hypothesized but not yet empirically demonstrated. The distinctions above are theoretical and await empirical testing (Table 3).

**Table 3:** Hypothesized discriminant boundaries and proposed empirical tests.

Construct pair	Hypothesized discriminant boundary	Proposed empirical test	Falsification criterion
Allostatic Integrity (AI) vs. Allostatic Load (AL)	AI captures adaptive capacity (higher = better); AL captures cumulative damage (higher = worse). Should correlate negatively (*r* ≈ −0.3 to −0.5) but not be identical.	Confirmatory factor analysis: bifactor model (AI and AL as separate factors) must fit better than unifactorial model (CFI *Δ* > 0.01, RMSEA Δ > 0.015).	If unifactorial model fits equally or better (ΔCFI < 0.01)
Allostatic Integrity vs. Cognitive Reserve (CR)	AI includes peripheral systems (physiology, epigenetics, interoception); CR is neural/cognitive. Should show moderate correlation (r ≈ 0.2–0.4) but dissociation in high-pathology samples.	Structural equation models: AI should predict general health outcomes (e.g., mortality); CR should specifically predict cognitive performance (e.g., MMSE).	If AI does not predict general health or CR does not predict cognitive performance
Allostatic Integrity vs. Frailty Index (FI)	AI includes dynamic regulation (recovery, synchrony); FI is cumulative deficit. AI should be more sensitive to interventions (changes in weeks/months); FI changes slowly (months/years).	Longitudinal study with repeated measures (baseline, 3, 6, 12 months): AI should show greater intra-individual variability and response to interventions (ICC AI < ICC FI).	If AI does not show greater temporal variability than FI
Allostatic Integrity vs. Resilience (traditional)	Resilience is outcome (successful adaptation); AI is the proposed system that produces that outcome. AI should precede and predict resilience in longitudinal designs.	Mediation analysis: AI should mediate the relationship between adversity and health outcomes (significant indirect effect, bootstrapped 95% CI excludes 0).	If indirect effect is not significant

### 4.9 Operational definition of allostatic integrity (hypothesized)

Allostatic integrity is proposed as a latent variable that can be operationalized through five domains (presented here as conceptual categories, not empirically weighted):

**Table:** 

Domain	Proposed markers
1. Adaptive reserve	Heart rate variability (high-frequency power), cortisol awakening response, DHEA/cortisol ratio
2. Inflammatory resilience	IL-10/IL-6 ratio, CRP (<3 mg/L), TNF-α
3. Multi system synchrony	Correlation between epigenetic age and physiological stress markers
4. Interoceptive accuracy	Heartbeat detection task accuracy, insula connectivity
5. Circular recovery	Speed of return to physiological baseline after acute stress challenge

*Operational definition of circular recovery*: Speed of return to baseline following an acute stressor (e.g., Trier Social Stress Test), operationalized as the half-life (t½) of cortisol, heart rate, and inflammatory cytokines (IL-6, TNF-α) from peak to baseline (Kirschbaum et al., 1993). Faster recovery (shorter t½) indicates higher allostatic integrity.

*Addressing colinearity*: We acknowledge that domains are not independent (e.g., HRV and cortisol recovery are correlated). The composite index should be derived empirically (e.g., as a latent variable) rather than as a simple sum. The domains listed are conceptual categories, not statistically orthogonal factors.

*Explicit caveat*: The Allostatic Integrity Index described here is purely illustrative and hypothesizing. It should not be used for clinical decision-making pending empirical validation in prospective cohorts and intervention trials.

## 5 Applying the model to specific disease contexts

### 5.1 Alzheimer’s disease

Neuroinflammation is now recognized as a core driver of AD pathogenesis, potentially preceding and exacerbating amyloid and tau pathology (Heneka et al., 2015). Our model hypothesizes that individuals with low allostatic integrity will show faster progression from mild cognitive impairment to dementia for any given level of neuroinflammation. Conversely, individuals with high allostatic integrity may tolerate substantial neuroinflammation without clinical decline (Jack et al., 2000).

*Disease-specific mechanism*: Allostatic load × APOE4 interaction: APOE4 carriers may show stronger effects of allostatic load on microglial activation (Verduzco Espinoza et al., 2025).

*Disease-specific prediction*: Allostatic integrity will predict conversion from MCI to AD dementia independently of amyloid burden (Ruesga-Mundo, 2026b).

*Emerging preventive strategies*: Recent observational evidence has highlighted the potential of live-attenuated herpes zoster vaccination as a candidate intervention for dementia prevention, with consistent findings across multiple natural experiments suggesting a reduction in dementia risk. However, the field now urgently requires large-scale randomized trials to definitively establish causality and quantify the effect size (Geldsetzer, 2026). From the perspective of the Hierarchical-Circular Model, such a vaccine could exert its effects through multiple levels—modulating peripheral immune responses (Level 4), reducing systemic inflammation and allostatic load (Level 3), and potentially influencing epigenetic or interoceptive pathways—thereby reinforcing the model’s emphasis on multidomain, systems-level interventions.

### 5.2 Major depressive disorder

The inflammatory hypothesis of depression posits that pro-inflammatory cytokines influence monoamine metabolism, neuroendocrine function, and synaptic plasticity (Miller and Raison, 2016). Our model extends this by proposing that allostatic integrity moderates the depression-inflammation relationship. Treatment-resistant depression may represent a state of chronically low allostatic integrity, potentially requiring multidomain interventions (lifestyle, psychotherapy, anti-inflammatory agents) rather than monoamine targeting alone.

*Disease-specific mechanism*: Interoceptive dysfunction in MDD may impair recognition of inflammatory states, delaying help-seeking and treatment response.

*Disease-specific prediction*: Patients with MDD and low allostatic integrity will show poorer response to standard antidepressants but good response to combined antidepressant + lifestyle intervention.

### 5.3 Schizophrenia

Prenatal stress and maternal immune activation are established risk factors for schizophrenia (Estes and McAllister, 2016; Ruesga-Mundo et al., 2025). Our model interprets these findings through epigenetic embedding: prenatal inflammatory signals may induce lasting DNA methylation changes in genes regulating microglial function and synaptic pruning. The heterogeneity of schizophrenia phenotypes may reflect different patterns of circular disruption across Levels 1–5.

*Disease-specific mechanism*: Prenatal epigenetic marks in stress-related genes may predict later microglial dysregulation and altered synaptic pruning.

*Disease-specific prediction*: First-episode psychosis patients with low allostatic integrity will show poorer recovery and higher relapse rates, independent of antipsychotic treatment (Ruesga-Mundo, 2026a).

### 5.4 Multiple sclerosis and other autoimmune conditions

In multiple sclerosis (MS), peripheral autoimmune activity and central neuroinflammation are tightly coupled. Our model hypothesizes that allostatic integrity—specifically the capacity for vagal regulation of inflammation—modulates relapse rates and progression. Interventions that enhance allostatic integrity (stress reduction, exercise, heart rate variability biofeedback) may complement immunomodulatory therapies.

## 6 Testable predictions

A central requirement of any scientific hypothesis is that it generates concrete, falsifiable predictions. Our model yields four core predictions that differentiate it from linear, single-pathway models.

### 6.1 Prediction 1: composite indices of allostatic integrity will outperform single biomarkers

*Rationale*: If chronic neuroinflammation emerges from disruptions in circular information flow, a composite measure capturing this integrated state should predict outcomes more accurately than any single biomarker.

*What makes this prediction unique*: The model predicts that dyssynchrony profiles (e.g., high inflammation with low cortisol, indicating feedback loop failure) will have the strongest predictive power—not merely the sum of individual markers.

*Testable approach*: Construct composite indices integrating Level 2 (DNA methylation at FKBP5, NR3C1), Level 3 (allostatic load markers), Level 4 (cytokine profiles, HPA axis measures), and Level 5 (interoceptive accuracy). Compare AUC values for predicting transition to chronic neuroinflammation.

*Outcome*: Transition to chronic neuroinflammation (operationalized as elevated IL-6, TNF-*α*, and CRP for ≥6 months + neuroimaging evidence).

*Design*: Longitudinal cohort (N ≥ 500, 3-year follow-up).

*Statistical model*: ROC AUC comparison (DeLong test).

*Falsification*: If single biomarker AUC > composite AUC (*p* < 0.05).

### 6.2 Prediction 2: multidomain interventions will have multiplicative (synergistic) effects

*Rationale*: Because levels are coupled through bidirectional feedback loops, interventions targeting multiple levels should produce synergistic—not merely additive—effects.

*What makes this prediction unique*: The model predicts a statistically significant positive interaction (multiplicative effect). An additive effect (non-significant interaction) would indicate that the levels operate independently, contradicting the model’s core assumption of circular causality.

*Testable approach*: 2 × 2 factorial RCT (allostatic integrity enhancement intervention vs. direct anti-inflammatory agent vs. combination vs. placebo). Dependent variable: reduction in neuroinflammatory markers (e.g., IL-6, TNF-α).

*Statistical model*: Two-way ANOVA with intervention A × intervention B interaction term.

*Falsification criterion*: The model would be contradicted if, in a well-powered study (≥80% power to detect a moderate interaction, *f* ≥ 0.25), the interaction term is non-significant (*p* > 0.05) and the effect size is consistent with additivity (interaction *η*^2^ < 0.01). A non-significant interaction in an underpowered study would not constitute falsification.

*Clarification*: A significant interaction (*p* < 0.05) with a positive coefficient (combination effect > sum of individual effects) supports the model. A significant negative interaction (antagonism) would also contradict the model’s prediction of synergy.

### 6.3 Prediction 3: allostatic integrity moderates inflammation-clinical outcome relationships

*Rationale*: Allostatic integrity may determine the clinical impact of neuroinflammation. In linear models, inflammation and outcome are often assumed monotonic. Our model predicts a statistical crossover interaction: high allostatic integrity flattens the inflammation decline slope; low allostatic integrity steepens it.

*Testable approach*: Stratify individuals by both neuroinflammatory burden (e.g., CSF/plasma cytokines, neuroimaging) and allostatic integrity indices. Compare clinical trajectories over follow-up.

*Design*: Longitudinal with baseline measures of both neuroinflammation and allostatic integrity.

*Statistical model*: Linear mixed models with inflammation × allostatic integrity interaction term.

*Falsification criterion*: The model would be contradicted if, in a well-powered study (≥80% power to detect a moderate interaction, *f* ≥ 0.25), the interaction term is non-significant (*p* > 0.05) and the effect size is consistent with parallel slopes (η^2^ < 0.01). A significant interaction (*p* < 0.05) with opposite signs for slopes (i.e., high AI flattens the slope; low AI steepens it) supports the model. A significant interaction with the same sign slopes (e.g., both positive but different magnitudes) would also contradict the model’s specific prediction of a crossover pattern. A non-significant interaction in an underpowered study would not constitute falsification.

### 6.4 Prediction 4: improvements in allostatic integrity will reduce neuroinflammation independent of direct anti-inflammatory therapies

*Rationale*: If allostatic integrity is a systems property, interventions that enhance it (lifestyle modification, stress reduction, interoceptive training) should reduce neuroinflammation even without directly targeting inflammatory pathways.

*Testable approach*: Randomized controlled trial comparing an allostatic integrity enhancement intervention (e.g., mindfulness-based stress reduction + exercise + sleep optimization) to a direct anti-inflammatory agent and to combination therapy.

*Prediction*: The allostatic integrity intervention will show effects comparable to or greater than the direct anti-inflammatory agent, with multiplicative effects in combination.

*Statistical model*: ANCOVA with baseline inflammation as covariate.

*Falsification*: If anti-inflammatory agent alone outperforms allostatic integrity intervention (p < 0.05).

## 7 Implications for research and clinical practice

### 7.1 Biomarker development (hypothetical)

Current biomarker strategies focus on single molecules (CRP, IL-6). Our model suggests that composite indices capturing allostatic integrity may have greater predictive validity. We propose a provisional, conceptual Allostatic Integrity Index comprising five domains (no weights assigned pending empirical derivation):

**Table:** 

Domain	Proposed markers
Adaptive reserve	HRV (high frequency), cortisol awakening response, DHEA/cortisol ratio
Inflammatory resilience	IL-10/IL-6 ratio, CRP (<3 mg/L), TNF-α
Multi-system synchrony	Correlation between epigenetic age and physiological stress markers
Interoceptive accuracy	Heartbeat detection task accuracy, insula connectivity
Circular recovery	Speed of return to baseline after acute stress challenge

*Explicit caveat*: Weights are omitted pending empirical derivation; the domains are conceptual categories only. The Allostatic Integrity Index described here is purely illustrative and hypothesizing. It should not be used for clinical decision-making pending empirical validation.

### 7.2 Multidomain prevention (hypothetical)

If allostatic integrity determines vulnerability to chronic neuroinflammation, prevention strategies might target multiple levels simultaneously:

#### 7.2.1 Level 1: genetic architecture and morphogenetic programming

Although genetic factors are not directly modifiable, their influence can be modulated through gene–environment interactions. Early identification of high-risk genetic variants (e.g., APOE4, IL-6, TNF-α polymorphisms, FKBP5) could inform personalized prevention strategies. Interventions that reduce environmental stress, optimize nutrition, and promote healthy lifestyle behaviors may attenuate the expression of pro-inflammatory genetic predispositions (Binder, 2009; Verduzco Espinoza et al., 2025). Additionally, emerging epigenetic therapies and nutrigenomic approaches may offer future avenues for modulating genetic risk (Feng et al., 2025).

#### 7.2.2 Level 2: epigenetic molecular memory

Interventions that reverse maladaptive epigenetic marks (diet, exercise, stress reduction, pharmacological agents) (Feng et al., 2025; Ruesga-Mundo et al., 2026).

#### 7.2.3 Level 3: allostatic load and systemic physiological adaptation

Reduction of allostatic load through metabolic, cardiovascular, and inflammatory management (Ruan et al., 2025; Souza-Talarico et al., 2025).

#### 7.2.4 Level 4: PINE network regulation

Psychoneuroimmuneendocrine network regulation (sleep optimization, social connection, psychotherapy, stress management) (Dantzer et al., 2008; Slavich and Irwin, 2014).

#### 7.2.5 Level 5: interoceptive-neuronal integration

Interoceptive training to enhance awareness and regulation of internal states (mindfulness, biofeedback, heartbeat detection training) (Barrett and Simmons, 2015; Khalsa et al., 2018; Hazelton et al., 2025).

Additionally, emerging evidence suggests that immunomodulatory interventions such as live-attenuated herpes zoster vaccination may represent a promising multidomain preventive strategy, potentially acting through both direct antiviral and indirect anti-inflammatory mechanisms (Geldsetzer, 2026). From the perspective of the Hierarchical-Circular Model, such interventions may exert effects across multiple levels—modulating peripheral immune responses (Level 4), reducing systemic inflammation and allostatic load (Level 3), and potentially influencing epigenetic or interoceptive pathways (Levels 2 and 5)—thereby reinforcing the model’s emphasis on systems-level, multidomain interventions. Large-scale randomized trials are urgently needed to confirm these observational findings and to establish the role of such vaccines within a comprehensive, systems-level prevention framework (Geldsetzer, 2026).

### 7.3 Personalized treatment (hypothetical)

Treatment could hypothetically be tailored based on an individual’s allostatic integrity profile, but this requires clinical trials. Individuals with high allostatic integrity may tolerate neuroinflammation without aggressive anti-inflammatory therapy. Those with low allostatic integrity might benefit from interventions targeting the specific levels where their system is most dysregulated:

- Level 1 (Genetic): Pharmacogenomic guidance based on APOE4, IL-6, or FKBP5 genotypes.
- Level 2 (Epigenetic): Epigenetic-modifying agents or lifestyle interventions.
- Level 3 (Allostatic): Metabolic, cardiovascular, and inflammatory management.
- Level 4 (PINE): Psychotherapy, stress reduction, immunomodulation.
- Level 5 (Interoceptive): Biofeedback, mindfulness, interoceptive training.

This personalized, systems-level approach would be delivered in addition to any direct anti-inflammatory agents, with the specific combination determined by each individual’s pattern of dysregulation across the five levels.

## 8 Limitations and future directions

Several limitations must be acknowledged.

First, the model’s strength—its integration of multiple levels—is also a source of complexity. Testing its predictions requires multi-modal data, sophisticated analytical approaches, and large sample sizes. However, the model is explicitly designed to generate falsifiable predictions using existing cohorts.

Second, much of the evidence synthesized is correlational. Establishing causality requires intervention studies that manipulate specific levels and observe effects across the system. Prediction 2 (multiplicative effects of multidomain interventions) provides a strong test.

Third, the model was developed with neuroinflammation as the focus, but its principles may extend to other domains. Future work should test whether allostatic integrity predicts outcomes across inflammatory conditions beyond the central nervous system.

Fourth, operationalization of allostatic integrity requires empirical validation. The provisional index proposed above must be tested across diverse populations.

Fifth, potential moderators not fully addressed in this framework include sex/gender differences, socioeconomic status, ethnic/racial factors, and prior treatment history. These should be incorporated in future empirical tests.

## 9 Conclusion

The hierarchical-circular model of biological memory offers a hypothesis-generating framework for understanding chronic neuroinflammation across neurodegenerative and neuropsychiatric diseases. By reframing neuroinflammation not as a linear cascade but as a potential failure of allostatic integrity—the hypothesized capacity for adaptive circular information flow across genetic, epigenetic, physiological, PINE network, and interoceptive levels—the model suggests possible resolutions to key paradoxes and generates falsifiable predictions for empirical testing.

The central construct of allostatic integrity, if validated, could serve as a dynamic systems-level property that moderates the relationship between inflammatory pathology and clinical expression. The four core predictions derived from the model provide clear opportunities for empirical testing using existing longitudinal cohorts and intervention trials.

If empirically supported, the model may eventually offer a conceptual basis for biomarker development, multidomain prevention, and personalized treatment strategies. At present, it is best understood as an integrative hypothesis intended to generate further research.

## Data Availability

The original contributions presented in the study are included in the article/supplementary material, further inquiries can be directed to the corresponding author.
